# Typhlitis in a Non-Neutropenic Patient: A Clinical Case

**DOI:** 10.7759/cureus.91552

**Published:** 2025-09-03

**Authors:** Pauline Kaisin, Jason Kasongo, Fabien Guerisse

**Affiliations:** 1 Emergency Department, Centre Hospitalier Universitaire (CHU) Charleroi-Chimay, Charleroi, BEL

**Keywords:** abdominal pain, cecum, ileocecal syndrome, neutropenic enterocolitis, typhlitis

## Abstract

Typhlitis, also known as neutropenic enterocolitis, is an inflammatory condition primarily affecting the cecum. It is most commonly associated with neutropenia, particularly in patients undergoing chemotherapy or with other forms of immunosuppression. However, rare cases have been described in non-neutropenic individuals.

A 53-year-old patient presented to the emergency department with right lower quadrant abdominal pain evolving over the past 24 hours. On admission, the patient was afebrile. Laboratory findings showed no evidence of neutropenia. Abdominal imaging led to the diagnosis of typhlitis, despite the absence of typical predisposing factors.

This case highlights that typhlitis can occur outside the classical context of neutropenia. Early recognition of this condition, even in immunocompetent patients, is essential to initiate appropriate management and prevent complications such as intestinal perforation, which remains the primary prognostic factor.

## Introduction

Typhlitis, also known in the literature as neutropenic enterocolitis or ileocecal syndrome, is an inflammation localized to the cecum, colon, or small intestine that primarily occurs in neutropenic patients, although some cases have been described in non-neutropenic individuals [1-3]. The main risk factors associated with the development of typhlitis are immunosuppressive conditions such as chemotherapy, AIDS, and other causes of acquired immunodeficiency [2]. Diagnosis relies on laboratory tests and imaging studies [1]. Supportive care is the primary approach; however, surgical intervention may become necessary in cases complicated by bowel perforation [1,4]. This clinical case presents an example of typhlitis in a non-neutropenic patient, illustrating the importance of considering this diagnosis when evaluating abdominal pain, as well as the diagnostic approach involved.

## Case presentation

A 53-year-old man undergoing hemodialysis five times a week via a right arm arteriovenous fistula presented to the emergency department. He was referred by his general practitioner for acute right lower quadrant abdominal pain that began the previous day. The pain had a sudden onset and was associated with one episode of vomiting and diarrhea.

The patient’s medical history included stage IV chronic kidney disease requiring dialysis, secondary to a familial nephropathy, arterial hypertension, previous varicocele surgery, and pyloromyotomy. There was no history of immunosuppressive medication use, and HIV infection was ruled out. He has no history of alcohol or tobacco use and was allergic to *Hymenoptera* venom.

Upon admission, his temperature was noted to be 37.5°C. His vital signs included blood pressure of 123/85 mmHg, heart rate of 88 beats per minute, and oxygen saturation of 98% on room air. The patient had a body mass index of 35 kg/m².

Physical examination revealed localized pain and peritoneal guarding in the right lower quadrant. Aside from the presence of a right arm arteriovenous fistula, the remainder of the clinical examination was unremarkable.

**Figure 1:**
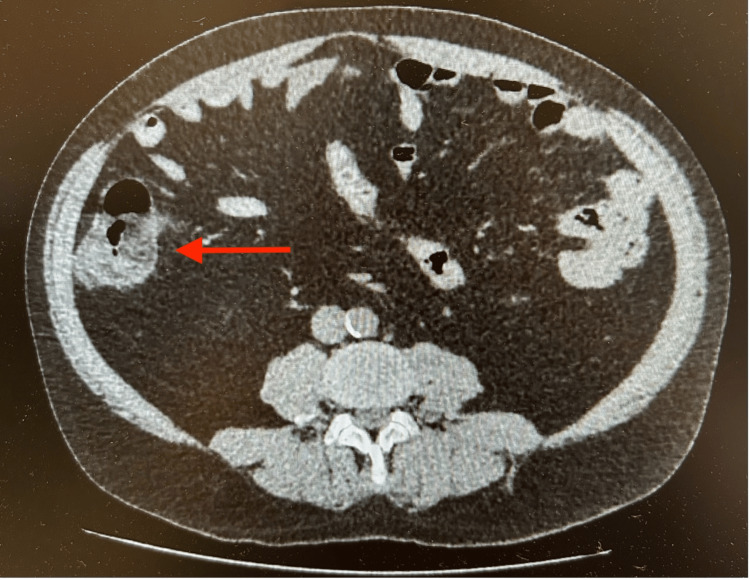
Contrast-enhanced computed tomography of the abdomen showing focal inflammatory involvement of the cecum (red arrow), with pericecal fat stranding.

An abdominal computed tomography (CT) scan was performed on the day of admission and showed focal inflammatory involvement of the cecum with wall thickening and surrounding fat stranding, sparing the terminal ileum and appendix, with no signs of perforation, pneumoperitoneum, or abscess formation (Figure 1).

The patient was hospitalized for 48 hours in the nephrology unit. During his stay, he continued his scheduled hemodialysis sessions and was started on empirical antibiotic therapy with ornidazole. Due to the lack of improvement in inflammatory markers, the regimen was switched after 24 hours to cefuroxime and metronidazole. This combination was chosen to provide appropriate coverage for the suspected pathogens and was guided by local antibiotic resistance patterns. It was continued for a total of seven days.

The diagnosis of typhlitis was made based on clinical findings and imaging, which also helped to rule out appendicitis. Blood tests and a repeat abdominal CT scan were performed on day 2, showing regression of the pericecal fat stranding and a decrease in biological inflammatory markers.

## Discussion

Typhlitis, most commonly seen in neutropenic patients, is a severe complication that arises following chemotherapy with agents known to damage the intestinal mucosa [1-4]. It can also occur in patients who are immunosuppressed for other reasons, such as HIV/AIDS, anti-rejection therapies, or congenital/acquired immunodeficiencies [2-5].

The chemotherapeutic agents associated with this complication include cytarabine, etoposide, daunomycin, doxorubicin, methotrexate, vincristine, irinotecan, alemtuzumab, taxane-based chemotherapy, and prednisone [1,3,6]. In terms of epidemiology, the condition is more frequently observed in children than in adults and it was initially described in pediatric leukemia patients [1,2]. Current literature estimates its incidence between 0.4% and 6% in pediatric oncology populations [1]. There is no significant difference in prevalence between male and female patients [6,7]. It is more often associated with hematologic malignancies than with solid tumors [1].

The pathogenesis of typhlitis remains poorly understood. It appears to begin with a breach in the intestinal mucosa, which then allows for bacterial translocation, leading to intramural infection and edema [1,2]. These changes cause wall thickening and induration visible on imaging [1]. The disease can progress to mucosal ulceration and necrosis, eventually resulting in intestinal perforation and sepsis [1,4,8].

Chemotherapy slows mucosal turnover, potentially triggering this cascade [1,2,4]. Another theory suggests local invasion by malignant cells may initiate the process [2]. Neutropenia increases the risk of infection due to the body’s inability to counteract bacterial translocation [2,4].

The most affected sites are the cecum, appendix, and terminal ileum [1,3,4]. Because of its localization and inflammation, the condition is often mistaken for appendicitis [1].

According to a 2021 study, the pathogens most frequently associated with progression to sepsis or septic shock are *Clostridium septicum*, *Citrobacter freundii*, *Stenotrophomonas maltophilia*, and *Stomatococcus mucilaginosus* [2]. Common clinical symptoms include abdominal pain, typically in the right lower quadrant, which may be accompanied by fever, diarrhea, or vomiting [1,4]. However, these symptoms are non-specific, contributing to diagnostic delays and misdiagnosis (such as appendicitis) [1,8].

The diagnosis relies primarily on clinical evaluation and imaging. Typhlitis is characterized by bowel wall thickening greater than 4-5 mm on cross-sectional imaging, along with inflammatory changes [1,3]. While abdominal ultrasound may be used, CT scanning is the preferred modality [1,3,4].

CT findings may include pneumatosis intestinalis, fat stranding, and ileus and wall thickening [1,3,4,8]. It also plays a crucial role in detecting complications such as bowel perforation and monitoring disease progression [1].

The definitive diagnosis is histological, showing edema, mucosal ulceration, and necrosis without inflammatory infiltrate, in contrast to appendicitis, where neutrophilic infiltration of the wall is typical [1,5]. Endoscopy is often contraindicated due to the high risk of perforation [5].

Histological confirmation allows differentiation from other differential diagnoses, such as acute appendicitis, inflammatory bowel disease, ischemic colitis, *Clostridioides difficile* pseudomembranous colitis, appendiceal abscess, and graft-versus-host disease [5,9].

Treatment is primarily supportive, with surgical intervention recommended in the event of complications such as bowel perforation [1,4]. In oncology patients, mortality rates range from 2.2% to 48%, with prognosis largely dependent on the degree of neutropenia and the extent of bowel wall thickening on CT [1,5,6]. A systematic review by Nematolahi et al. showed that antibiotic prophylaxis is associated with decreased mortality, especially in immunocompromised patients, whereas anti-fungal prophylaxis was associated with higher mortality [6].

As suggested above, data on typhlitis in non-neutropenic patients are scarce. Diagnosis still relies heavily on imaging, and it may occur in the absence of neutropenia [8].

In this case, chronic kidney disease requiring long-term dialysis can be considered a potential risk factor. As demonstrated in multiple studies, patients with end-stage kidney disease undergoing chronic dialysis often present with immune dysfunction, related to both the accumulation of uremic toxins and the dialysis modality itself [10-12].

A case similar to the one described here was reported in 2021, involving a young woman with no identifiable immunosuppressive condition who presented with a clinical picture suggestive of appendicitis [8]. Although the patient in that report was immunocompetent, unlike the patient discussed here who exhibited immune dysfunction related to chronic kidney disease, several common features were observed in both cases: CT imaging confirmed the diagnosis and supportive antibiotic therapy was successfully employed [8].

In most cases, mortality is associated with cecal perforation [5]. Typhlitis in non-neutropenic individuals remains exceedingly rare. However, the absence of neutropenia does not equate to a fully functional immune system, and several hypotheses have been proposed to explain the pathogenesis of typhlitis in otherwise immunocompetent individuals [8].

## Conclusions

Typhlitis is a rare condition, particularly in non-neutropenic patients. It must nonetheless be recognized due to the high risk of complications associated with diagnostic delay. Diagnosis relies on clinical assessment, imaging, and, when available, histological confirmation. The absence of neutropenia does not exclude typhlitis, especially in immunocompromised individuals. Given its often non-specific presentation, imaging plays a pivotal role in establishing the diagnosis.

Prognosis is closely tied to the risk of cecal perforation. While management is typically conservative and, as in this case, may result in a favorable outcome with antibiotic therapy, complications necessitate urgent surgical intervention. It is also essential to investigate potential underlying causes of immunosuppression in such patients. In this case, chronic kidney disease likely contributed to the patient’s immune dysfunction.
